# A deep-learning approach to predict mortality in very-low-birth-weight infants treated in the intensive care units using DeepSurv

**DOI:** 10.3389/fped.2026.1801840

**Published:** 2026-07-28

**Authors:** Nishankul Bozhbanbayeva, Arailym Abilbayeva, Anel Tarabayeva, Iskander Isgandarov, Talgat Tolykbayev, Indira Adilbekova, Dinara Yelyubayeva

**Affiliations:** 1 Neonatology Department, Asfendiyarov Kazakh National Medical University, Almaty, Kazakhstan; 2 Shortanbayev General Immunology Department, Asfendiyarov Kazakh National Medical University, Almaty, Kazakhstan; 3 Laboratory of Breeding and Biotechnology, Institute of Plant Biology and Biotechnology, Almaty, Kazakhstan; 4 Zhambyl Regional Perinatal Center, Taraz, Kazakhstan; 5 Department of Highly Dependent Newborns (Intensive Care), The Center for Perinatology and Pediatric Cardiac Surgery, Almaty, Kazakhstan

**Keywords:** DeepSurv, machine learning, mortality, prediction, very-low-birth-weight infants

## Abstract

**Background and objectives:**

Predicting mortality in very low-birth-weight (VLBW) infants remains a critical challenge in neonatology. While traditional scoring systems and standard machine learning models are widely used, they often fail to capture the complex, non-linear interactions and time-dependent dynamics of neonatal clinical courses. This study addresses these limitations by evaluating DeepSurv to enhance predictive accuracy and clinical risk stratification in the neonatal intensive care unit. This study aimed to thoroughly compare the performance of the DeepSurv model with that of traditional machine learning models, such as Cox proportional hazards (CoxPH) and random survival forest (RSF), including temporal metrics.

**Materials and methods:**

A retrospective analysis of 958 VLBW neonates was performed using data from an integrated health information system from January 2021 to May 2025. The Boruta algorithm, a feature selection method that identifies statistically significant predictors by comparing their importance scores with those of random, shadow variables, identified 11 such predictors in our study.

**Results:**

DeepSurv demonstrated superior discriminatory ability with an overall AUC of 0.95, substantially outperforming RSF (AUC of 0.89) and CoxPH (AUC of 0.86). The temporal AUC analysis also favored DeepSurv. Moreover, DeepSurv recorded the lowest Brier scores (IBS 0.071) throughout the entire follow-up period compared with CoxPH (IBS 0.129) and RSF (IBS 0.126). Calibration curves showed acceptable agreement between predicted and observed risk by day 28 for all models, whereas at day 7 calibration was weaker, particularly for DeepSurv at low predicted-risk values, and Kaplan–Meier analysis, along with log-rank tests, demonstrated their effectiveness in risk stratification. Decision curve analysis revealed that the DeepSurv model had the highest clinical utility at both day 7 and day 28, underscoring its potential impact on neonatal care.

**Conclusion:**

DeepSurv outperformed traditional models in predicting VLBW mortality, demonstrating superior clinical utility and robustness against overfitting. While the early neonatal period (days 5–7) remains the most challenging to predict, DeepSurv provides a powerful framework for personalized risk stratification, though further optimization is needed to capture the highly dynamic changes of the first week of life.

## 1 Introduction

The issue of neonatal mortality remains one of the most pressing challenges in modern healthcare, especially among very low–birth-weight (VLBW) infants. Despite significant progress in neonatology over recent decades, mortality rates in this group remain high in many countries (1, 2). The increased risk of both death and illness in newborns is closely linked to low birth weight and low gestational age (3).

VLBW newborns are at high risk of developing numerous complications, such as respiratory distress syndrome (RDS), intraventricular hemorrhage (IVH), necrotizing enterocolitis (NEC), infectious diseases, and other pathological conditions that can lead to death (4). Accurate and timely prediction of mortality risk is therefore of particular scientific and clinical interest within neonatology.

In routine clinical practice, various scoring systems are commonly used to evaluate neonatal mortality risk, including the neonatal critical illness score (NCIS), the neonatal therapeutic intervention scoring system (NTISS), the Clinical Risk Index for Babies (CRIB) and its revised version CRIB-II, the score for neonatal acute physiology with perinatal extension (SNAPPE) and its revised version SNAPPE-II, and the score for neonatal acute physiology (SNAP) and its revised version SNAP-II (5). However, studies confirm the lower efficiency of these scores compared to machine learning models (6–8). This is because most of these scoring systems rely on limited physiological parameters, which can lead to the loss of important prognostic information. Additionally, their methodological foundation often assumes linear relationships between predictors and outcomes, along with an additive effect of various factors. In reality, pathophysiological interactions are frequently nonlinear, and the effects of individual factors may be complex and nonadditive. These limitations highlight the need for more advanced analytical methods capable of capturing complex, nonlinear dynamics.

Recent advances in machine learning (ML) methods have shown significant promise across various medical fields, especially in predicting clinical outcomes (9). Current research actively explores the potential of machine learning to predict neonatal mortality in diverse infant populations, with reported areas under the ROC curve (AUC) values that vary widely from 0.58 to 0.97 (10, 11). However, a critical analysis of existing studies reveals two major methodological and translational gaps. First, most studies focus almost exclusively on standard aggregate performance metrics and often rely on binary outcomes at fixed time points. This approach fails to account for time-to-death information and does not examine how the model's predictive accuracy evolves over the clinical course. Second, there is a lack of studies translating the obtained prognostic estimates into differentiated, practically applicable risk categories for individual patients, which hinders more effective use of models to optimize clinical decision-making and personalize therapeutic strategies.

The rise of deep learning (DL) methods in survival analysis in recent years has led to significant methodological advances (12). In 2018, Katzman et al. introduced DeepSurv, a personalized treatment recommendation system based on deep learning with a multilayer neural network (13). Since then, the DeepSurv model and its variants have shown strong performance in survival analyses across various oncological diseases (14–18), liver cirrhosis (19), COVID-19 (20), and in identifying risk factors for diabetes, hypertension, and dyslipidemia (21). However, when DeepSurv was applied to pediatric cohorts, only one study focused on examining the socioeconomic characteristics of mortality in children under 5 years of age in African countries (22). To date, no studies have evaluated DeepSurv in very low–birth–weight (VLBW) neonates within neonatal intensive care units (ICUs).

This study aims to compare the performance of the DeepSurv model with that of traditional machine learning algorithms and classic survival models in predicting neonatal mortality in VLBW infants treated in the neonatal intensive care unit. A key feature of our approach is the inclusion of a detailed, time-dependent performance analysis, along with the development of practical tools for patient risk prediction and stratification.

## 2 Materials and methods

### 2.1 Data source and study population

This study was reported in accordance with the TRIPOD + AI (Transparent Reporting of a multivariable prediction model for Individual Prognosis or Diagnosis plus Artificial Intelligence) reporting guideline for clinical prediction models using regression and machine learning methods. A completed TRIPOD + AI checklist is provided in the Supplementary Material.

The data for this retrospective cohort study were collected from neonatal intensive care units at the Perinatology and Pediatric Cardiac Surgery Center, City Perinatal Center No. 3, the Scientific Center of Obstetrics, Gynecology, and Perinatology in Almaty, and the Zhambyl Regional Perinatal Center in Taraz, which includes information about VLBW infants. Data were obtained for the period from January 2021 through May 2025 via the integrated Damumed Health Information System (https://alm.dmed.kz).

Initially, 1,106 records were collected. Data harmonization was performed by concatenating records from four independent sources. During this process, all variables were standardized to a common format, and the representation of time-related variables was unified to ensure consistency across datasets. Newborns with a birth weight above 1,500 g and those with severe congenital malformations were excluded. To minimize bias, we decided to use the complete case exclusion method. This approach was chosen because the proportion of excluded records was less than 5% of the total dataset. The primary outcome was death during the observation period. Time-to-event was defined as the number of days from birth to death. Infants who were transferred from the NICU prior to the end of the observation period were treated as censored at the date of transfer. Records with at least one missing value in the variables were excluded from the final dataset. After removing records with missing data, 958 of the original 1,106 eligible medical records remained for analysis. The study protocol was approved by the Ethics Committee of S.D. Asfendiyarov Kazakh National Medical University (Protocol No. 8 (144), November 3, 2023). We confirm that all methods used adhere to current guidelines and regulations, and that this study was conducted in strict accordance with the ethical principles of the Declaration of Helsinki. Informed consent was obtained from each participant.

### 2.2 Study variables and grouping

To develop a prognostic model for mortality prediction in VLBW neonates, an expanded set of clinical variables was collected for each patient admitted to the neonatal ICU. The demographic and clinical–laboratory characteristics of the VLBW cohort are detailed in Supplementary Table S1. Vital signs, including heart rate and oxygen saturation, were recorded as single-point values obtained immediately after birth at the time of NICU admission, as part of routine neonatal clinical assessment. The diagnostic status of respiratory distress syndrome, intraventricular hemorrhage, necrotizing enterocolitis, and disseminated intravascular coagulation was recorded as a binary present/absent variable reflecting whether the condition had been confirmed by the time of clinical assessment, rather than being restricted to findings present strictly at the moment of NICU admission. Because these conditions may develop at variable points during the neonatal course rather than being present from birth, a “Yes” status could correspond to a diagnosis established either at admission or later during the NICU stay. To identify the most important predictors, the Boruta algorithm was employed. Boruta acts as a wrapper around the random forest classifier. Boruta provides stable and unbiased feature selection (23). Feature selection was performed using the training set only to avoid information leakage; the selected predictors were then applied unchanged to the held-out test set. The random forest framework offers metrics of feature importance and captures interactions among variables.

### 2.3 Dataset splitting

**Figure 1:**
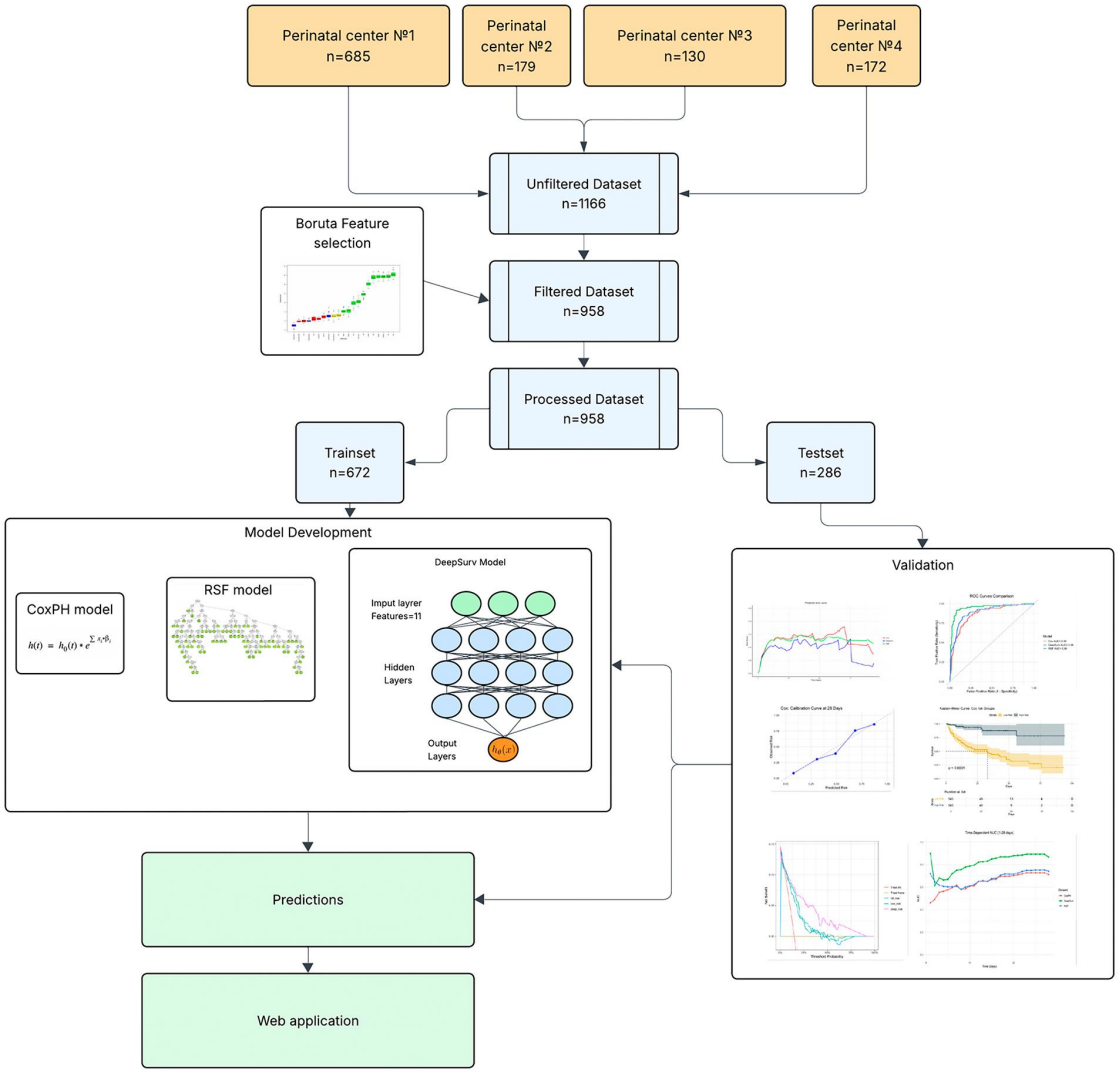
Process of data preparation.

To ensure an objective assessment of predictive performance and prevent overfitting, the dataset was divided into training and test sets at a 7:3 ratio. The split was performed at the patient level using a random allocation procedure. The training set was used to develop and train the models, whereas the test set was utilized to evaluate the models' predictive performance. A flowchart in Figure 1 illustrates the process of data preparation.

### 2.4 Review and justification of model selection

In this study, three survival models were developed and compared: the Cox proportional hazard model (CoxPH), the Random Survival Forest (RSF), and the DeepSurv deep learning model. All three models were implemented as survival models, aiming to predict time-to-event.

The Cox proportional hazards (CoxPH) model is a semiparametric regression model that estimates the effect of covariates on the hazard function without requiring specification of the baseline hazard. It assumes that the hazard ratio between any two individuals is constant over time (24). The model expresses the hazard as:

$$h(t|X)=h_{0}(t)\exp(\beta X)$$

where $h_{0}(t)$ is the baseline hazard, *X* is the vector of covariates, and β are the regression coefficients. This structure enables straightforward interpretation of effect sizes in terms of hazard ratios.

In contrast, the RSF model is an ensemble tree-based approach that combines multiple decision trees. Each tree is built using log-rank splitting to optimize the separation of survival curves. Because of its nonparametric nature, RSF is more flexible and resistant to outliers than parametric models (25).

DeepSurv is a deep neural network that uses a modified version of the Cox model architecture by replacing the linear predictor with a multilayer perceptron. The network is trained using a modified Cox partial likelihood loss. This makes it suitable for censored survival data (13).

### 2.5 Hyperparameter tuning

To achieve optimal predictive performance, a hyperparameter tuning process was conducted. The DeepSurv model was optimized via the Optuna framework. Hyperparameter tuning was performed through a 10-repeated random search with 5-fold cross-validation on the training dataset. The concordance index (C-index) was used to evaluate the models with different hyperparameter combinations. During this process, the sizes of the hidden layers, the dropout rate, the learning rate, and the L2 regularization coefficient were adjusted to reduce overfitting and enhance the model's predictive accuracy. For the RSF model, hyperparameter tuning was carried out via the built-in “tune()” function from the “randomForestSRC” package in R. Key parameters such as “mtry” and “nodesize” were optimized on the training set. The model was built with 1,000 trees, with the subsample size for each tree automatically selected. The optimization was guided by a predefined threshold for error function improvement and a set number of iterations, enabling the selection of a model with the best parameters. The final hyperparameters for the DeepSurv and RSF models are listed in Supplementary Table S2.

### 2.6 Model quality assessment

To assess the quality of the model predictions, we used various metrics and evaluation methods. Discriminative ability was evaluated with Harrell's concordance index (C-index) and receiver operating characteristic (ROC) curves, including the calculation of the area under the curve (AUC). ROC analysis was conducted at two complementary levels. Time-dependent ROC curves were computed at successive daily landmark times using inverse-probability-of-censoring weighting to capture how discrimination evolves over the follow-up period, and an overall AUC was computed as a single, time-independent ROC AUC over the full test cohort, comparing each model's continuous risk score against the binary mortality outcome, irrespective of time-to-event. In general, a C-index or AUC of 0.5 indicates no discriminative ability. Values ≥ 0.70 are generally considered acceptable and indicative of satisfactory discrimination. Accuracy and calibration were assessed via the Brier score (BS) and the integrated Brier score (IBS), as were calibration plots that visually illustrate the agreement between predicted and observed event probabilities. For survival data, an IBS <0.25 is often considered acceptable. To assess the variability of these metrics due to the limited test set size, we applied a bootstrap procedure with 500 resamples with replacement. For each bootstrap sample, C-index was recalculated, and 95% confidence intervals (CI) were derived using the percentile method.

The clinical utility and potential impact of each model on clinical decision-making were evaluated via decision curve analysis (DCA). Risk stratification on the basis of Kaplan–Meier survival curves and the log-rank test was employed to categorize patients into low-risk and high-risk groups. For each model, patients in the test set were split at the median of that model's predicted risk score, with scores above the median classified as high predicted survival and scores at or below the median classified as low predicted survival. This fixed, data-driven 50/50 split, rather than an optimized or clinically pre-specified threshold, accounts for the balanced group sizes.

Finally, Shapley Additive exPlanations (SHAP) analysis was used to interpret and understand the impact of individual features on the DeepSurv model's predictions. All performance metrics were evaluated on the test set, while the C-index was additionally assessed for both the training and test sets.

### 2.7 Web application

Using the best-performing model, we developed a bilingual web application to enable individualized, point-of-care estimation of survival probability for very-low-birth-weight infants treated in the neonatal ICU. The tool is intended for use both at the time of NICU admission and for serial reassessment as the patient's clinical status evolves during the NICU stay. At admission, the diagnostic status of disseminated intravascular coagulation (DIC), IVH, NEC, and RDS should be entered as confirmed at that time. If any of these conditions is subsequently diagnosed during hospitalization, the tool should be re-applied with the updated status to generate a clinically current survival estimate rather than relying solely on the admission-time prediction. The application generates an individualized survival probability curve over a 100-day observation period and provides specific probability estimates at Day 7 and Day 28. The application was implemented in Python using the Streamlit framework (version 1.43.2).

### 2.8 Statistical analysis

To examine the potential differences in patient characteristics between the training and testing data, the *t*-test and the Mann–Whitney *U* test were performed to compare continuous variables. The chi-square (*χ*^2^) test was used to analyze categorical variables. All the statistical analyses were conducted via R software (version 4.4.2) and Python (version 3.13.2). A two-sided *p*-value of <0.05 was considered statistically significant.

## 3 Results

### 3.1 Population characteristics

In the present study, data from 958 preterm VLBW neonates were analyzed. Of the 958 included infants, 244 deaths (25.5%) were recorded during the observation period, and 714 infants were censored. In the training cohort (*n* = 672), 161 deaths and 511 censored observations were recorded; in the test cohort (*n* = 286), 83 deaths and 203 censored observations were recorded. The main demographic, anthropometric, and clinical characteristics of the study population, along with their distributions between the training and test cohorts with statistical tests, are shown in Supplementary Table S3. Most of the variables were evenly distributed between the two cohorts, except for two variables that showed a statistically significant between-cohort difference. In particular, intrauterine growth restriction was more prevalent in the training cohort than in the test cohort (27% vs. 19%, *p* = 0.005), and the 5-minute Apgar score differed between the two cohorts (*p* = 0.046). The average gestational age of the neonates in the overall cohort was 29 ± 3 weeks, and the average birth weight was 1,107 ± 305 g. The sex distributions were roughly equal.

### 3.2 Feature selection

**Figure 2:**
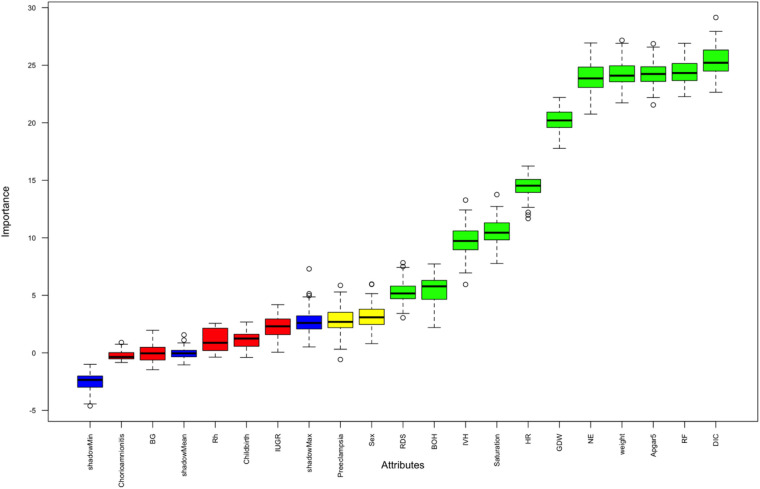
Significant variables determined by the boruta algorithm.

The Boruta algorithm was employed to identify the most relevant prognostic variables. Boruta selected 11 predictors, which were then used to develop the predictive models. The key variables included: bad obstetric history (BOH), birth weight, Apgar score at 5 min, gestational age (GA), presence of respiratory failure (RF), heart rate (HR), oxygen saturation level, presence of respiratory distress syndrome (RDS), intraventricular hemorrhage (IVH), disseminated intravascular coagulation (DIC) syndrome, and necrotizing enterocolitis (NEC). A graphical display of the analysis results is shown in Figure 2.

### 3.3 Model performance

**Figure 3:**
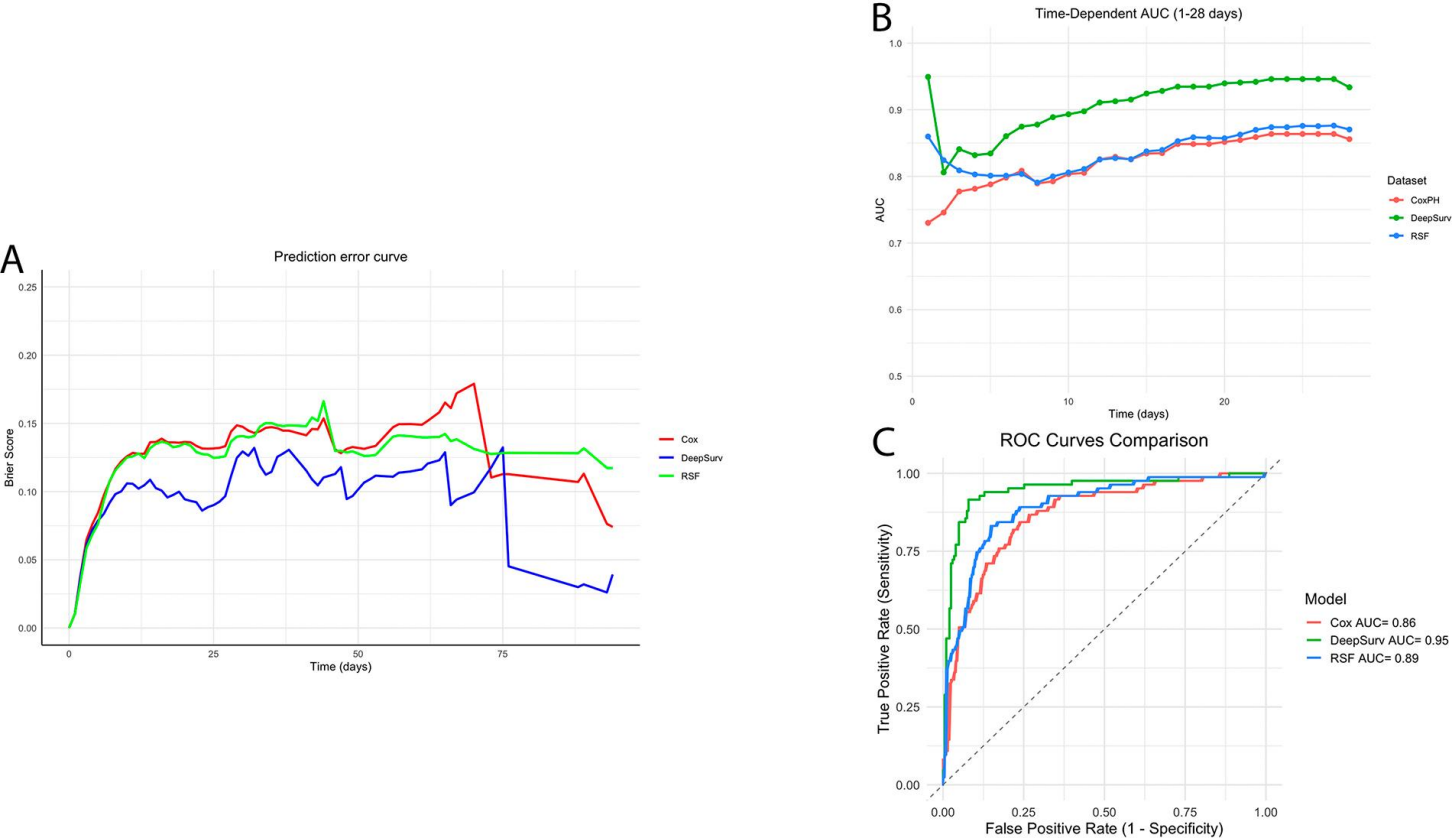
Quality evaluation metrics for the models (test set). **(A)** Prediction error curves (Brier score) over time for the models. **(B)** Time-dependent ROC curve analysis of the models. **(C)** Generalized ROC curve for the models.

After hyperparameter tuning and model training, the predictive performance of the models was evaluated. The Brier score was used as a key metric to assess prediction accuracy. All the developed models achieved Brier score values less than 0.25, indicating high predictive accuracy (Figure 3A). Compared with the other models, the DeepSurv model demonstrated the lowest prediction error, consistently yielding the lowest Brier score values across nearly all time points throughout the follow-up period.

With respect to discriminative ability, as measured by the concordance index (C-index), DeepSurv also achieved the highest values. For the test dataset, DeepSurv reached a C-index of 0.86 (95% CI: 0.81–0.90), whereas for the training dataset, it reached 0.94 (95% CI: 0.93–0.96). In contrast, the CoxPH model had a C-index of 0.81 (95% CI: 0.80–0.82) for the training set and 0.76 (95% CI: 0.75–0.77) for the test set. The RSF model had a C-index of 0.95 (95% CI: 0.94–0.96) for the training set and 0.78 (95% CI: 0.72–0.80) for the test set.

To assess the discriminative performance of the developed models, ROC analysis was conducted, including both time-dependent ROC curves and overall ROC curves for the test set (Figures 3B,C). The results showed that the DeepSurv model achieved the highest area under the curve (AUC) value of 0.95 (95% CI: 0.91–0.98). This model was followed by the RSF model with an AUC of 0.89 (95% CI: 0.84–0.93), whereas the CoxPH model had the lowest AUC of 0.86 (95% CI: 0.83–0.92).

A detailed analysis of the time-dependent ROC curves (Figure 3B) and their associated AUC values revealed that, although DeepSurv generally had better discriminative ability, there was a slight and clinically insignificant decline in the AUC during the first 5 days after birth.

Notably, all the AUC values for the analyzed models exceeded 0.80, indicating strong effectiveness in predicting survival.

**Figure 4:**
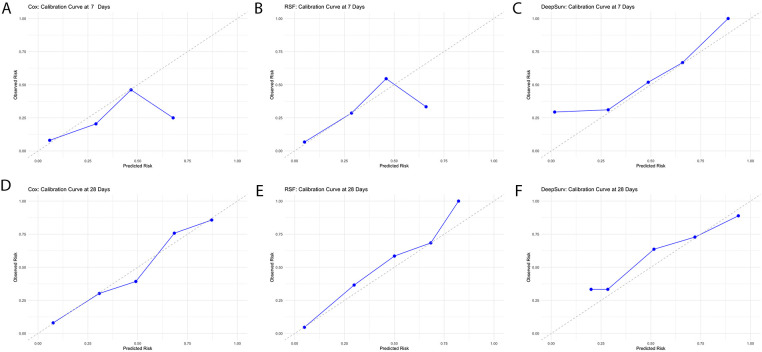
Calibration curve analysis (test set). **(A)** Calibration curve for the CoxPH model on day 7. **(B)** Calibration curve for the RSF model on day 7. **(C)** Calibration curve for the DeepSurv model on day 7. **(D)** Calibration curve for the CoxPH model on day 28. **(E)** Calibration curve for the RSF model on day 28. **(F)** Calibration curve for the DeepSurv model on day 28.

Calibration plots were created at two time points—day 7 and day 28 are displayed in Figures 4A–F. Calibration plots at day 7 and day 28 showed time-dependent variability in agreement between predicted and observed risks across models, with improved agreement by day 28.

The calibration curve for the CoxPH model on day 7 (Figure 4A) shows that at a predicted risk of approximately 0.05, the observed risk is approximately 0.08. At a predicted risk of 0.28, the observed risk reaches 0.22. The highest observed risk of approximately 0.45 corresponds to a predicted risk near 0.45, followed by a decrease in observed risk to 0.25 at a predicted risk of 0.65. On day 28 (Figure 4D), the CoxPH model aligns more closely with the ideal calibration line, with an observed risk of 0.1 at a predicted risk of 0.08, which increases to 0.85 at a predicted risk of 0.88.

Similarly, the calibration curve for the RSF model on day 7 (Figure 4B) showed a trend similar to that of the CoxPH model. On day 28 (Figure 4E), the RSF also exhibited good calibration, with most points closely aligned with the diagonal line.

For the DeepSurv model, the calibration curve on day 7 (Figure 4C) shows that at a predicted risk of approximately 0.05, the observed risk is approximately 0.28. At a predicted risk of 0.28, the observed risk remains steady at 0.29. The observed risk then increases to 0.52 at a predicted risk of 0.48, then to 0.68 at a predicted risk of 0.65, and finally reaches 1.0 at a predicted risk of 0.9. On day 28 (Figure 4F), the DeepSurv model also exhibited acceptable calibration performance.

**Figure 5:**
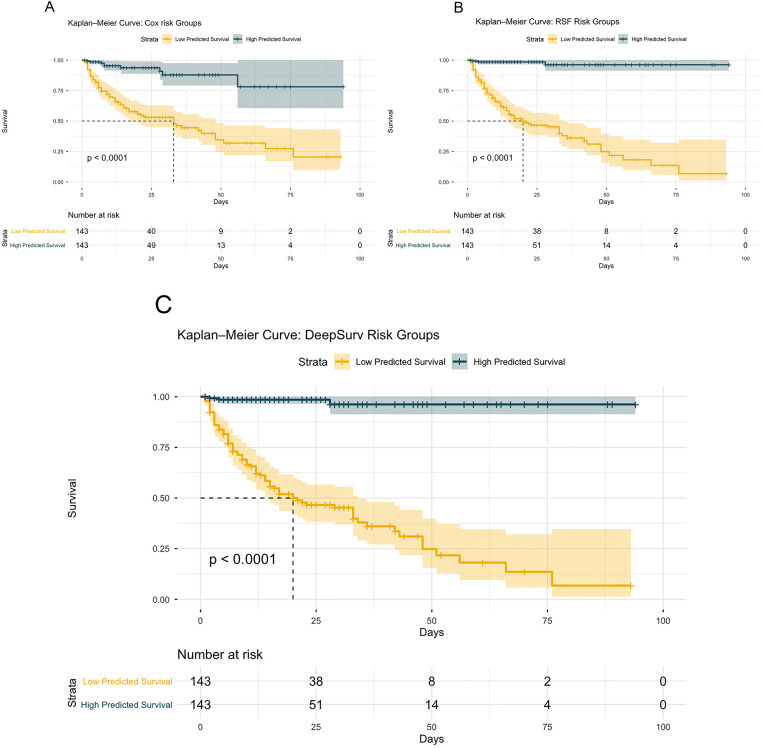
Kaplan–Meier survival curves and log-rank test results for mortality risk stratification in VLBW neonates on the basis of model predictions (test set). **(A)** CoxPH model. **(B)** RSF model. **(C)** DeepSurv model.

For the risk stratification task across VLBW infants, Kaplan–Meier survival curves were created for the CoxPH, RSF, and DeepSurv models. Each model started with 143 patients in both groups, stratified by predicted survival probability (high predicted survival vs. low predicted survival). For the CoxPH model (Figure 5A), by day 25, 49 patients remained alive in the high predicted survival group, and 40 remained alive in the low predicted survival group; the log-rank test revealed a statistically significant difference between the groups (*p* < 0.0001). Similarly, for the RSF model (Figure 5B), 51 survivors remained in the high predicted survival group, and 38 remained alive in the low predicted survival group by day 25 (*p* < 0.0001). In the case of the DeepSurv model (Figure 5C), 51 patients survived in the high predicted survival group, and 38 survived in the low predicted survival group by day 25 (*p* < 0.0001).

Overall, both the log-rank test results and visual analysis of the Kaplan–Meier curves clearly show that all three developed models effectively stratify mortality risk.

**Figure 6:**
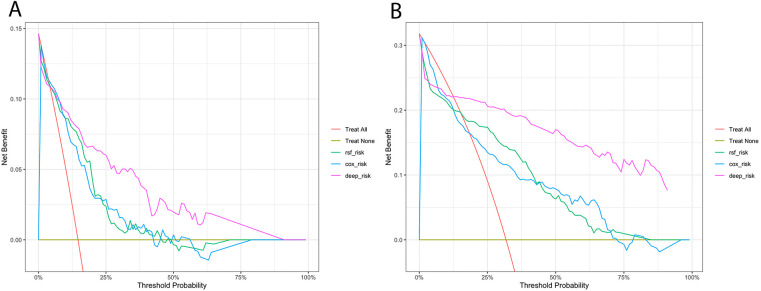
Decision curve analysis for assessing the clinical utility of the models (test set). **(A)** On day 7. **(B)** On day 28.

The clinical usefulness of the models was evaluated via decision curve analysis (DCA) at two time points: day 7 and day 28. The DCA results are shown in Figure 6. The analysis revealed that the predictions of the DeepSurv model provided the highest net clinical benefit across a range of threshold probabilities, demonstrating superior clinical utility.

### 3.4 DeepSurv model interpretation

**Figure 7:**
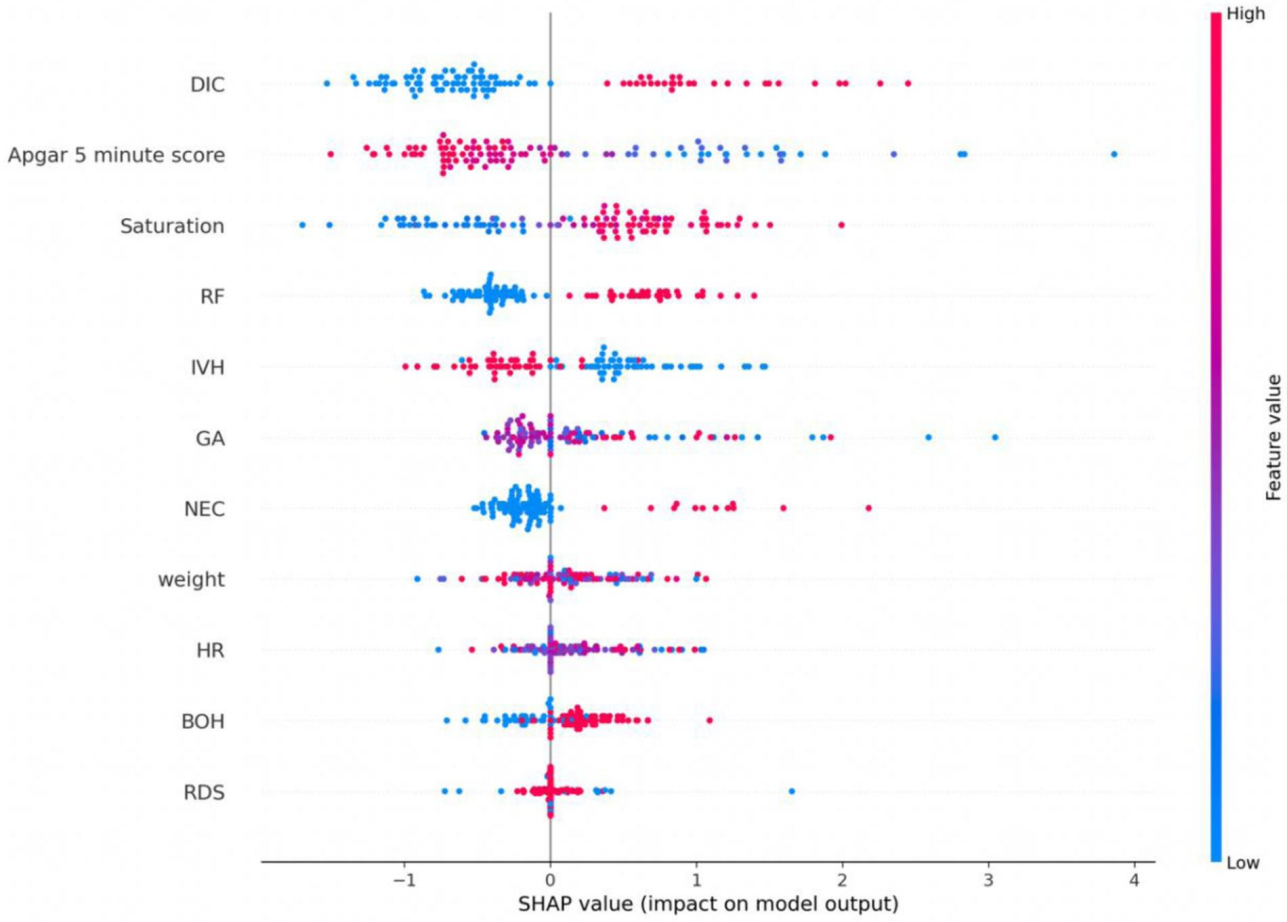
SHAP analysis for the DeepSurv model.

To improve the interpretability of the DeepSurv model and understand how individual features contribute to the predicted survival risk, SHAP analysis was conducted. A SHAP summary plot was created to visually display both the relative importance and the directional influence of each input variable on the model's predictions (Figure 7). Importantly, the high contribution of DIC identified in the SHAP analysis should be interpreted not as its predictive strength strictly at baseline, but rather as the strong association of evolving coagulopathy with mortality during the critical observation period.

### 3.5 Web application

**Figure 8:**
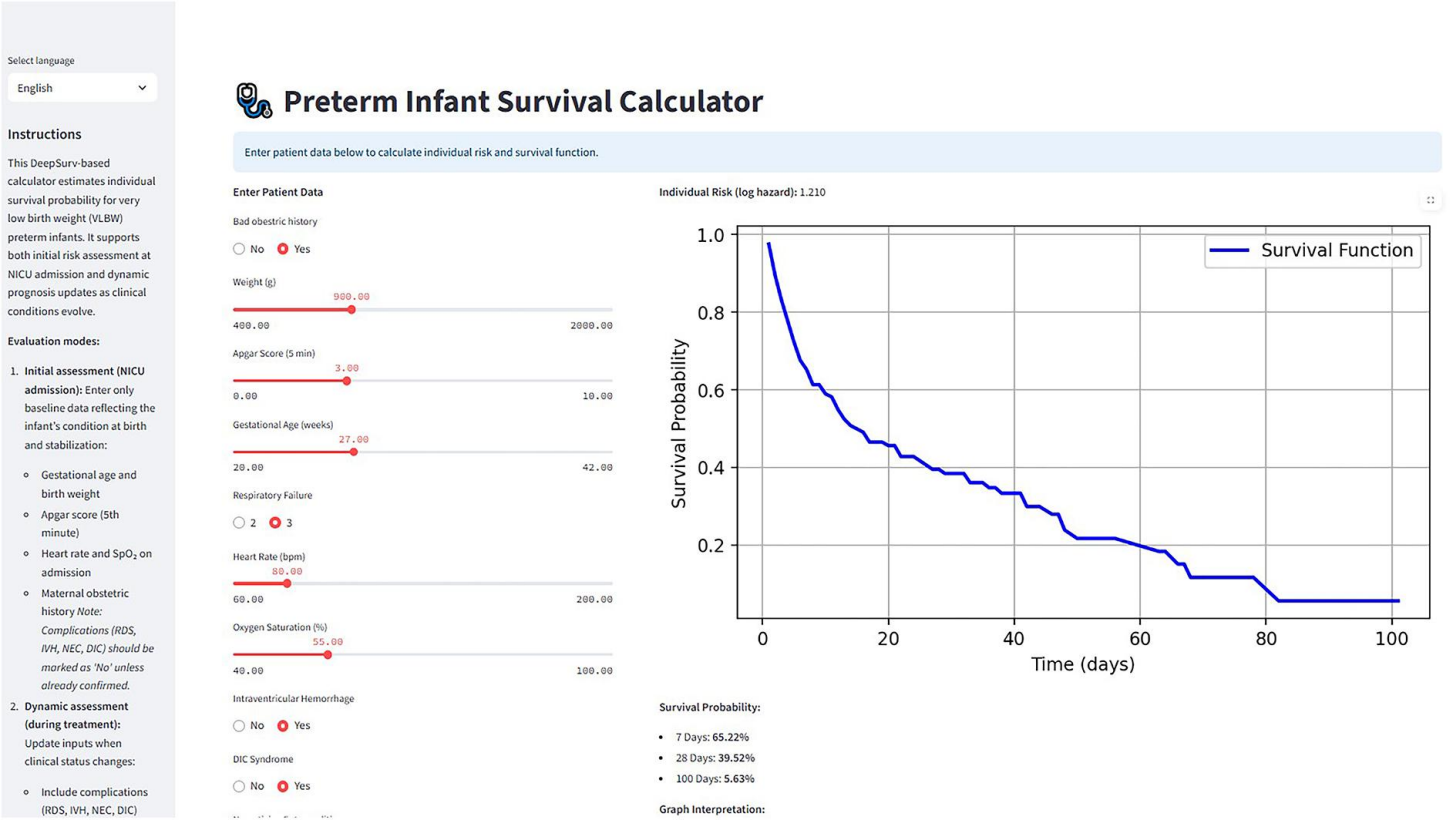
Interface of a DeepSurv-based web tool for individual survival prediction.

Based on the DeepSurv model, we developed a web application for individualized prediction of survival probability in VLBW infants admitted to the neonatal ICU (Figure 8). Because the diagnostic status of DIC, IVH, NEC, and RDS may evolve over the course of hospitalization, the tool is designed to be re-applied whenever the status of these conditions' changes, so that the survival estimate can be updated longitudinally rather than reflecting only a single, fixed admission-time assessment. The results are presented as a survival probability curve over a 100-day period, where a value of 1 indicates 100% survival probability and 0 indicates 0% survival probability. Additionally, the web tool provides estimated relative survival probabilities specifically for days 7 and 28. The web tool is available in two languages. The application can be accessed at the following link: (https://neomortalsurv.net) (Supplementary Table S4).

## 4 Discussion

As mentioned earlier, timely prediction of mortality in VLBW neonates is crucial for making informed and objective clinical decisions and optimizing treatment plans. Our study results demonstrate the high prognostic value of the DeepSurv model. This emphasizes the real potential for applying such personalized predictions, an area that has been previously limited by traditional methods.

In this study, we aimed to perform a comparative analysis of the prognostic performance of DeepSurv, a deep learning-based method, against two well-established survival analysis models. The CoxPH model was chosen as the traditional linear prediction model because of its interpretability and status as a standard reference in the survival analysis field. To represent a nonlinear approach, we included the RSF model, which has demonstrated high prognostic performance in neonatal populations, as reported in a recent publication (26). This approach enables complex analysis and helps position the DeepSurv model in relation to both traditional and advanced nonlinear predictive models within a specific cohort of neonates.

To ensure the broad applicability of the prognostic models, we conducted an analysis of publicly available clinical predictors that are routinely included in standard neonatal care protocols. The data were collected through a retrospective review by Damumed, an integrated healthcare information system widely used in Kazakhstan. From this database, information on VLBW preterm neonates and their associated clinical parameters recorded in the neonatal ICU was extracted.

To identify the best subset of predictors for mortality prediction in VLBW preterm neonates, the Boruta algorithm was used (23). A comparison of different survival models revealed that the DeepSurv model had the highest discriminative ability, with an overall AUC of 0.95. The RSF and CoxPH models also performed well, with AUC values of 0.89 and 0.86, respectively.

An analysis of time-dependent prognostic efficiency, observed through the time-dependent AUC, confirmed the consistent dominance of the DeepSurv model in predicting mortality among preterm neonates over most of the 28-day follow-up period. It significantly outperformed both the classical CoxPH model and the RSF model. A brief but noncritical decline in the time-dependent AUC for DeepSurv was noted during the first five days after birth. We attribute this to the increased sensitivity of the model architecture to the pronounced physiological instability and substantial clinical heterogeneity characteristic of the early neonatal period in this specific cohort of VLBW infants (27, 28). The physiological systems of VLBW neonates are extremely immature and highly susceptible to decompensation even under minimal stressors, leading to conditions such as neonatal sepsis, necrotizing enterocolitis, and a wide range of respiratory, cardiovascular, neurological, and other complications. These conditions are typically associated with significant variability in both laboratory and instrumental data. Additionally, the intensive therapeutic interventions administered to this vulnerable population can further contribute to clinical parameter instability (29). The hypothesis that DeepSurv models have increased sensitivity to noisy input data is supported by previously published findings (30).

Analysis of calibration curves revealed certain diagnostic challenges for all studied survival models at day 7 of observation. These results correlate with dynamic AUC values and are due to the specific nature of the early neonatal period. We believe that the observed calibration deviation is determined by the highly stochastic nature of clinical processes and the nonlinear progression of acute postnatal complications, such as fulminant sepsis and grade III–IV intraventricular hemorrhage. The pathophysiological manifestation of these conditions is often characterized by rapid dynamics, outpacing the reflection of the corresponding markers in laboratory parameters recorded upon admission.

Such “information noise” in the early stages of hospitalization poses a serious challenge to predictive modeling, as static input data are not always able to fully verify the hyperdynamic trajectories of critical conditions. However, it is crucial to note that DeepSurv showed visually closer agreement between predicted and observed risk than the CoxPH and RSF models, particularly in the moderate- and high-risk segments. This observation is descriptive, as no formal statistical test of calibration differences between models was performed, and should be interpreted accordingly. This supports the hypothesis that deep learning architectures have a greater potential for approximating complex nonlinear interactions, even in the face of highly volatile clinical data.

By day 28 of observation, a significant improvement in calibration parameters was recorded in all models. This trend is likely due to physiological stabilization of newborns and the formation of more deterministic prognostic patterns after the acute adaptation phase. The obtained results highlight the need for further optimization of prognostic tools for the extra-early period. A promising direction appears to be the development of modified DeepSurv configurations integrating high-frequency streaming data for real-time monitoring, which will allow for more effective verification of transient changes in vital functions (31, 32).

Analysis of the Brier score curve and the integrated Brier score strongly suggests that the DeepSurv model has the best calibration and the lowest overall prediction error throughout the entire observation period. Therefore, despite a short-term dip in discriminatory power during the first days after birth, DeepSurv's excellent calibration and its high discriminatory ability in later intervals make it the best choice for clinical use. We attribute the decline in DeepSurv's performance, marked by instability in the Brier score during the late period, to a significant reduction in the number of patients still being observed and a decrease in recorded events by this time. This data sparsity causes statistical instability in the evaluation metrics.

### 4.1 Ability to generalize and clinical usefulness

In our study, the DeepSurv model showed the best generalization and predictive accuracy among all tested algorithms, with C-index values of 0.94 in the training cohort and 0.86 in the testing cohort. These findings establish DeepSurv as the most promising model for potential clinical application.

The moderate discrepancy observed between the training and test performances for DeepSurv, compared with the simpler CoxPH model, indicates some overfitting of the model. However, this aligns with the widely recognized trends and known challenges of deep learning models. Although deep neural networks have the potential to outperform other models, they require careful management to avoid overfitting. The tendency of deep learning models to overfit is a well-known issue actively studied in machine learning and computational biology (33–35).

Notably, the RSF model displayed an even larger train–test discrepancy than DeepSurv, with a C-index of 0.95 in the training set vs. 0.78 in the test set (gap of 0.17, compared with 0.08 for DeepSurv). This more pronounced overfitting is likely related to the tuned RSF hyperparameters, in particular the minimum terminal-node size, which permits the growth of highly granular trees with terminal nodes containing as few as a single observation. While this setting minimized the cross-validated prediction error on the training set during tuning, small node sizes are known to increase the variance of tree-based ensembles and predispose them to overfitting. We did not constrain nodesize to a larger, pre-specified value in the present analysis.

Furthermore, in our study, all three models successfully stratified risk with statistical significance (*p* < 0.0001 for all models), dividing patients into high-risk and low-risk groups, with the DeepSurv model showing the best discrimination. Decision curve analysis (DCA), which is used to evaluate the clinical utility of the prognostic models, indicated that DeepSurv offered the greatest net clinical benefit compared with the RSF and CoxPH models at both day 7 and day 28 of follow-up.

The practical benefit of this work lies in its potential integration into electronic medical record systems as an automated clinical decision support tool.

### 4.2 Limitations of this study

This study has several limitations. First, the cohort was restricted to VLBW infants, which may limit generalizability to other neonatal populations. Second, while the model demonstrated strong performance during internal validation, external validation in different hospitals is necessary to ensure transportability before clinical implementation. Third, due to restrictions on the electronic health record system, we could not verify the distribution of patients from the four centers across the training and test sets. Fourth, infants transferred from the NICU were treated as censored observations. We acknowledge the potential for informative censoring and the absence of a formal competing risks analysis. Incorporating such models remains a priority for future methodological research. Fifth, the training and test cohorts differed significantly on two variables. The proportion of infants with intrauterine growth restriction was higher in the training set than in the test set (27% vs. 19%, *p* = 0.005), and the distribution of Apgar score at 5 min also differed significantly between the two cohorts (*p* = 0.046). Because IUGR was not among the Boruta-selected predictors, it did not directly enter the models. Nevertheless, infants with growth restriction may follow distinct mortality trajectories that are only indirectly reflected through correlated variables, which represents a limitation for generalizability. The Apgar score at 5 min, by contrast, is one of the 11 Boruta-selected predictors used by all three models, so the observed imbalance in its distribution between cohorts may have contributed to the moderate discrepancy between training- and test-set performance reported above and should be taken into account when interpreting the external generalizability of our findings. Sixth, complete-case analysis was used, excluding fewer than 5% of otherwise eligible records. We did not formally test whether these data were missing completely at random, and it is possible that missingness was related to illness severity or other unmeasured factors, which could introduce a degree of selection bias not captured by our current analysis. Furthermore, our study did not incorporate high-volume, high-velocity streaming data, such as continuous physiological signals. Integrating these real-time data streams could significantly enhance the model's predictive accuracy and clinical utility, representing a primary direction for future research. Finally, for some diagnoses, precise temporal annotations were unavailable, which limits the interpretation of predictors strictly at the time of ICU admission.

## 5 Conclusion

DeepSurv shows the best overall predictive performance and clinical usefulness. The early neonatal period (first 5–7 days) remains the most critical period and is difficult to predict. Overfitting is a common issue, but DeepSurv handles it better than other complex models do. These observations highlight the need for further optimization or development of specialized predictive tools tailored to the earliest neonatal period. This may involve the use of alternative models or specific DeepSurv configurations that capture highly dynamic changes in the data.

## Data Availability

The raw data supporting the conclusions of this article will be made available by the authors, without undue reservation.
