# Supplementary material for: A deep-learning approach to predict mortality in very-low-birth-weight infants treated in the intensive care units using DeepSurv
Source: Front Pediatr. 2026 Jul 28;14:1801840. doi: 10.3389/fped.2026.1801840 (PMC13459046; doi:10.3389/fped.2026.1801840)
Supplement: Supplementary file 1 [file Datasheet1.zip › Supplementary files/Supplementary table S1.docx]

**Supplementary table S1.** Demographic and clinico-laboratory characteristics of neonates with very low birth weight

| **Category of variables** | **Variables** | **Description / Units of measurement** |
| --- | --- | --- |
| **Maternal and perinatal factors** | Mother's age | Years |
|  | The presence of burdened  obstetric history (BOH) in the medical history | Yes/No |
|  | The presence of preeclampsia in the medical history | Yes/No |
|  | The presence of chorioamnionitis in the medical history | Yes/No |
|  | The presence of intrauterine  growth retardation (IUGR) in the medical history | Yes/No |
|  | Childbirth | Natural birth/Cesarean section |
| **Neonatal characteristics** | Sex | m/f |
|  | Birth weight | Grams (g) |
|  | Gestational age (GA) | Weeks |
|  | Apgar score at 5 minutes | Scores (0-10) |
|  | Respiratory failure (RF) | Degree (1-3) |
|  | Heart rate (HR) | Beats per minute (bpm) |
|  | Oxygen saturation | Percentages (%) |
|  | Blood group (BG) | (O, A, B, AB) |
|  | Rhesus factor (Rh) | Positive/Negative |
| **Complications of the early neonatal period** | Presence of respiratory distress syndrome (RDS) | Yes/No |
|  | Presence of intraventricular hemorrhage (IVH) | Yes/No |
|  | Presence of disseminated intravascular coagulation  syndrome (DIC) | Yes/No |
|  | Presence of necrotizing enterocolitis (NEC) | Yes/No |
