# Supplementary material for: A deep-learning approach to predict mortality in very-low-birth-weight infants treated in the intensive care units using DeepSurv
Source: Front Pediatr. 2026 Jul 28;14:1801840. doi: 10.3389/fped.2026.1801840 (PMC13459046; doi:10.3389/fped.2026.1801840)
Supplement: Supplementary file 1 [file Datasheet1.zip › Supplementary files/Supplementary table S2.docx]

**Supplementary table S2.** Final hyperparameters for the DeepSurv and RSF models

| **Model** | **Hyperparameters** |
| --- | --- |
| **DeepSurv** | Input features: 11; Hidden layers: [511, 141, 127, 16]; Activations: ReLU;  BatchNorm: Yes; Dropout: [0.201, 0.496, 0.106, 0.28]; Output: 1; Optimizer: Adam; Learning rate: 0.0045; Weight decay: 0.00097; Batch size: 128; Epochs: 100 |
| **RSF** | ntree: 1000; nodesize: 1; mtry: 2; splitrule: bs.gradient; importance: permute; nsplit: 10; samptype: swr |
