# Supplementary material for: A deep-learning approach to predict mortality in very-low-birth-weight infants treated in the intensive care units using DeepSurv
Source: Front Pediatr. 2026 Jul 28;14:1801840. doi: 10.3389/fped.2026.1801840 (PMC13459046; doi:10.3389/fped.2026.1801840)
Supplement: Supplementary file 1 [file Datasheet1.zip › Supplementary files/Supplementary table S3.docx]

**Supplementary table S3.** General and clinical characteristics of the subjects

| **Characteristic** | **Overall**  N = 958*^1^* | **Train**  N = 672*^1^* | **Test**  N = 286*^1^* | **p-value***^2^* |
| --- | --- | --- | --- | --- |
| **BOH** | 594 (62%) | 406 (60%) | 188 (66%) | 0.14 |
| **Preeclampsia** | 298 (31%) | 210 (31%) | 88 (31%) | >0.9 |
| **Chorioamnionitis** | 55 (5.7%) | 36 (5.4%) | 19 (6.6%) | 0.5 |
| **IUGR** | 237 (25%) | 184 (27%) | 53 (19%) | 0.005 |
| **Childbirth** | 665 (69%) | 462 (69%) | 203 (71%) | 0.5 |
| **Sex** | 477 (50%) | 331 (49%) | 146 (51%) | 0.7 |
| **Birth weight, m±SD** | 1,107 ±  305 | 1,101 ± 307 | 1,121 ± 298 | 0.3 |
| **Apgar score at 5 minutes** | | | | 0.046 |
| 2 | 20 (2.1%) | 10 (1.5%) | 10 (3.5%) |  |
| 3 | 89 (9.3%) | 64 (9.5%) | 25 (8.7%) |  |
| 4 | 142 (15%) | 111 (17%) | 31 (11%) |  |
| 5 | 136 (14%) | 87 (13%) | 49 (17%) |  |
| 6 | 255 (27%) | 184 (27%) | 71 (25%) |  |
| 7 | 282 (29%) | 189 (28%) | 93 (33%) |  |
| 8 | 31 (3.2%) | 25 (3.7%) | 6 (2.1%) |  |
| 9 | 3 (0.3%) | 2 (0.3%) | 1 (0.3%) |  |
| **GA, m±SD** | 29 ± 3 | 29 ± 3 | 29 ± 3 | 0.4 |
| **RF** | | | | 0.2 |
| 1 | 15 (1.6%) | 13 (1.9%) | 2 (0.7%) |  |
| 2 | 512 (53%) | 351 (52%) | 161 (56%) |  |
| 3 | 431 (45%) | 308 (46%) | 123 (43%) |  |
| **HR** | 114 ± 27 | 114 ± 28 | 114 ± 27 | 0.9 |
| **Oxygen saturation** | 75 ± 14 | 75 ± 14 | 75 ± 14 | 0.9 |
| **BG** | | | | 0.9 |
| O (I) | 323 (34%) | 232 (35%) | 91 (32%) |  |
| A (II) | 282 (29%) | 195 (29%) | 87 (30%) |  |
| B (III) | 274 (29%) | 191 (28%) | 83 (29%) |  |
| AB (IV) | 79 (8.2%) | 54 (8.0%) | 25 (8.7%) |  |
| **Rh** | 915 (96%) | 636 (95%) | 279 (98%) | 0.069 |
| **RDS** | 777 (81%) | 535 (80%) | 242 (85%) | 0.085 |
| **IVH** | 490 (51%) | 341 (51%) | 149 (52%) | 0.8 |
| **DIC** | 382 (40%) | 261 (39%) | 121 (42%) | 0.4 |
| **NEC** | 139 (15%) | 97 (14%) | 42 (15%) | >0.9 |
