# Supplementary material for: A deep-learning approach to predict mortality in very-low-birth-weight infants treated in the intensive care units using DeepSurv
Source: Front Pediatr. 2026 Jul 28;14:1801840. doi: 10.3389/fped.2026.1801840 (PMC13459046; doi:10.3389/fped.2026.1801840)
Supplement: Supplementary file 1 [file Datasheet1.zip › Supplementary files/Supplementary table S4.docx]

**Supplementary table S4.** Web application description and manual for use

| **Description of the web application** | The application was built using the Streamlit framework and deployed on a secure cloud server. Clinicians should input eleven patient-specific variables. Then application computes the individualized survival curve over a 100-day period. Point estimates of relative survival probabilities are provided at day 7 and day 28, corresponding to clinically relevant milestones. Results are displayed in a graphical and numerical format, allowing immediate interpretation. The tool is designed for ease of use and accessibility: Two-language support (Russian and English); Intuitive input form with validation to minimize data entry errors. Output visualization includes a Kaplan–Meier-like curve generated for the individual patient alongside numerical probability values. |
| --- | --- |
| Manual for use | When creating prognoses for conditions such as disseminated intravascular coagulation, intraventricular hemorrhage, and necrotizing enterocolitis at the time of admission to the intensive care unit, the "YES" option should only be selected if the condition has already been documented and confirmed. If the diagnosis is not recorded, select the "NO" option. In cases where these conditions develop later, the calculator can be re-run with the "YES" option selected to refine the prognosis. The web application can be accessed at:  [[https://neomortalsurv.net](https://neomortalsurv.net/)] |
